# Prognosis and Immune Infiltration of Chromobox Family Genes in Sarcoma

**DOI:** 10.3389/fonc.2021.657595

**Published:** 2021-05-11

**Authors:** Jian Zhou, Ziyuan Chen, Ming Zou, Rongjun Wan, Tong Wu, Yingquan Luo, Gen Wu, Wanchun Wang, Tang Liu

**Affiliations:** ^1^ Department of Orthopedics, The Second Xiangya Hospital, Central South University, Changsha, China; ^2^ Hunan Key Laboratory of Tumor Models and Individualized Medicine, The Second Xiangya Hospital, Central South University, Changsha, China; ^3^ Department of Orthopedics, Brain Hospital of Hunan Province (The Second People’s Hospital of Hunan Province), Changsha, China; ^4^ Department of Respiratory and Critical Care Medicine, Xiangya Hospital, Central South University, Changsha, China; ^5^ National Clinical Research Center for Geriatric Disorders, Xiangya Hospital, Changsha, China; ^6^ Department of General Medicine, The Second Xiangya Hospital, Central South University, Changsha, China; ^7^ Clinical Medicine Eight-Year Program, Central South University, Changsha, China

**Keywords:** CBX family genes, prognosis, immune infiltration, sarcoma, survival

## Abstract

**Background:**

Chromobox family genes (CBXs) are known to play roles in numerous modifications of the chromatin in order to inhibit the transcription of target genes. CBXs have been shown to be expressed at high levels in many types of cancer and can also serve as a target gene for therapeutic purposes. However, little is known about the expression and prognostic value of CBXs in human sarcomas.

**Methods:**

The transcription level of CBXs was analyzed using the Oncomine dataset, and the differential expression of CBXs in sarcoma was reported by the Gene Expression Profiling Interactive Analysis (GEPIA) dataset. We also used the CCLE dataset to evaluate the expression of CBXs in a sarcoma cell line. The prognostic value of CBXs was analyzed using GEPIA and Kaplan–Meier analysis. In addition, the corrections between CBXs and their co-expressed genes were reported using Oncomine and GEPIA datasets. DAVID was used to perform GO function enrichment analysis for the CBXs and their co-expression genes. Finally, TIMER was used to analyze the immune cell infiltration of CBXs in patients with sarcoma.

**Results:**

HP1-*α/β/γ* (CBX1/3/5) and CBX4/6/8 were found to be overexpressed in human sarcoma, and CBXs were upregulated in almost all the sarcoma cell line. The expression levels of HP1-*α/β/γ* (CBX1/3/5) and CBX7 were associated with overall survival (OS) in patients with sarcoma, while high expression levels of CBX7 were related to disease-free survival (DFS). In addition, the expression levels of CBX2/6/7 were related to recurrence-free survival (RFS). We also found that the CBX family was positively correlated with the infiltration of immune cells, including CD8^+^ T cells, CD4^+^ T cells, B cells, macrophages, neutrophils, and dendritic cells, in sarcoma.

**Conclusions:**

The results from the present study indicated that CBXs were significantly associated with prognosis and immunological status in sarcoma. These data suggest that CBXs could serve as potential biomarkers for prognosis and immune infiltration in human sarcoma.

## Introduction

Sarcomas are rare but aggressive bone and soft tissue malignancies that afflict patients of all ages. Sarcomas are usually incurable because chemotherapy and surgery are not effective (1); these malignant tumors also exhibit broad differentiation (2). Although some causative factors have been established for sarcomas, including environmental factors, stimulation by foreign matter, and endocrine dyscrasia, the precise underlying causes of sarcoma have yet to be elucidated. Although generally rare, sarcomas can be found in patients of any age and tend to occur more commonly in adolescents and teenagers than in the elderly (3). Osteosarcoma is the most common form of primary bone sarcoma and accounts for 1% of all tumors; the incidence of osteosarcoma in children was previously reported to be five per million (4).

Chromobox family genes (CBXs) are associated with a variety of modifications to the chromatin that inhibit the transcription of target genes as key elements of polycomb repressive complex 1 (PRC1) (5). Currently, eight members of the CBX family have been identified, based on their single N-terminal chromosomal domains, consisting of three β folds and an α helix. There are two broad categories of CBX genes: heterochromatin protein 1 and polycomb complexes. HP1-*α/β/γ* (CBX1/3/5) are heterochromatic proteins while CBX2/4/6/7/8 are polycomb complexes. The heterochromatin protein 1 group consists of an N-terminal chromodomain and a C-terminal chromodomain, while the polycomb group contains only a conservative N-terminal chromodomain (6). Different CBX proteins have been associated with different parts of the chromatin, leading to the specific transcription of target genes (7, 8).

According to previous studies, CBXs are known to be involved in the occurrence and development of a diverse range of tumors through various pathways (9, 10). For example, Han et al. reported that CBX2 could act as a tumor promoter in osteosarcoma by targeting miRNA let-7a (11). In addition, Ma et al. reported that CBX3 was related to an unfavorable prognosis and tumorigenesis in patients with osteosarcoma (12). In another study, Wang et al. demonstrated that targeting the CK1*α*/CBX4 axis may provide benefit to patients with metastasis of osteosarcoma (13). Liang et al. found that HP1-*α/β/γ* (CBX1/3/5) and CBX2/4/6/7 exerted an effect on breast cancer, thus indicating that CBX2 is expressed at high levels in basal-like and HER-2 subtypes (14). In addition, CBX4/7 is highly expressed in Luminal A and Luminal B subtypes of breast cancer (14, 15). The increased mRNA expression of HP1-*β/γ* (CBX1/3) and CBX2 has been linked to a poorer Relapse free survival (RFS) *via* survival analysis; better outcomes were found to be associated with higher expression levels of CBX4/5/6/7 (14). Moreover, the high expression levels of HP1-*β/γ* (CBX1/3) were associated with overall survival (OS) and disease-free survival (DFS) in patients with non-small-cell lung cancer. Furthermore, the elevated expression of HP1-*γ* (CBX3) has been demonstrated to exert impact on tumor diameter and lymph node metastasis (16). A previous study reported that the increased mRNA expression of HP1-*β/γ* (CBX1/3) and CBX2/6/8 was correlated with a worse OS, while the overexpression of CBX7 was related to a greater OS in patients with hepatocellular carcinoma (17). However, the role of CBXs in sarcoma remains unclear. Here, we aimed to investigate the expression of CBX in sarcomas and the relationship between CBXs and prognosis/immune cell infiltration in patients with sarcoma.

## Methods

### Ethics Statement

This study was approved by the Second Xiangya Hospital of Central South University Committee for Clinical Research and all methods were carried out in accordance with the Declaration of Helsinki.

### ONCOMINE Analysis

The Oncomine database (https://www.oncomine.org/resource/login.html) is usually adopted to analyze DNA or RNA sequences from a comprehensive cancer microarray database and genome-wide expression data for malignant tumors. In the present study, we used the Oncomine database to identify the differential transcriptional expression of CBXs in multiple cancer tissues and corresponding normal tissues. Moreover, three databases including Detwiller sarcoma database (18), Barretina sarcoma database (19), and Quade uterus database (20) were used to analyze the differential transcriptional expression of CBXs in subtypes of sarcoma and corresponding normal tissues using p < 0.05 as the significance threshold.

### GEPIA Analysis

The GEPIA dataset (http://gepia.cancer-pku.cn/) is an online service that includes a spectrum of cancer expression data. The GEPIA dataset contains 9,736 tumor samples and 8,587 normal samples from the TCGA. The GEPIA dataset is usually used to analyze data arising from the TCGA project. In this study, we used the GEPIA database to analyze the expression levels of CBX genes in sarcoma tissues and normal tissues.

### CCLE Dataset Evaluation

The CCLE dataset (https://www.broadinstitute.org/ccle) is usually used to accurately depict the genetic characteristics of cancer cells. The CCLE dataset can provide information relating to DNA mutation and gene expression. We used the CCLE database to investigate the expression of CBXs in sarcoma cell lines.

### Kaplan–Meier Plotter Database Analysis

The Kaplan**–**Meier Plotter Database (https://kmplot.com/analysis/index.php?p=service&cancer=pancancer_rnaseq) is used to evaluate the influence of genes on the survival of patients afflicted with various forms of cancers. In this study, we used the Kaplan**–**Meier Plotter Database to analyze the relationship between the expression of CBXs in sarcoma and associated survival rates.

### TIMER Dataset Analysis

The TIMER database (https://cistrome.shinyapps.io/timer/) is an online service that can be used to investigate the infiltration of different immune cells and their clinical significance. In the present study, CBXs were input into the ‘Gene module’ tool of TIMER in order to generate scatterplots to investigate the association between CBX expression and immune infiltration in sarcoma.

### GO Enrichment Analyses of DEGs

The Database for Annotation, Visualization and Integrated Discovery (DAVID, http://david.ncifcrf.gov) (version 6.7), an online biological information database that provides a comprehensive set of functional annotation information for genes and proteins, was used to perform GO enrichment analyses of CBXs and their co-expression genes (21).

## Results

### Transcriptional Levels of the CBX Family in Patients With Sarcoma

**Figure 1:**
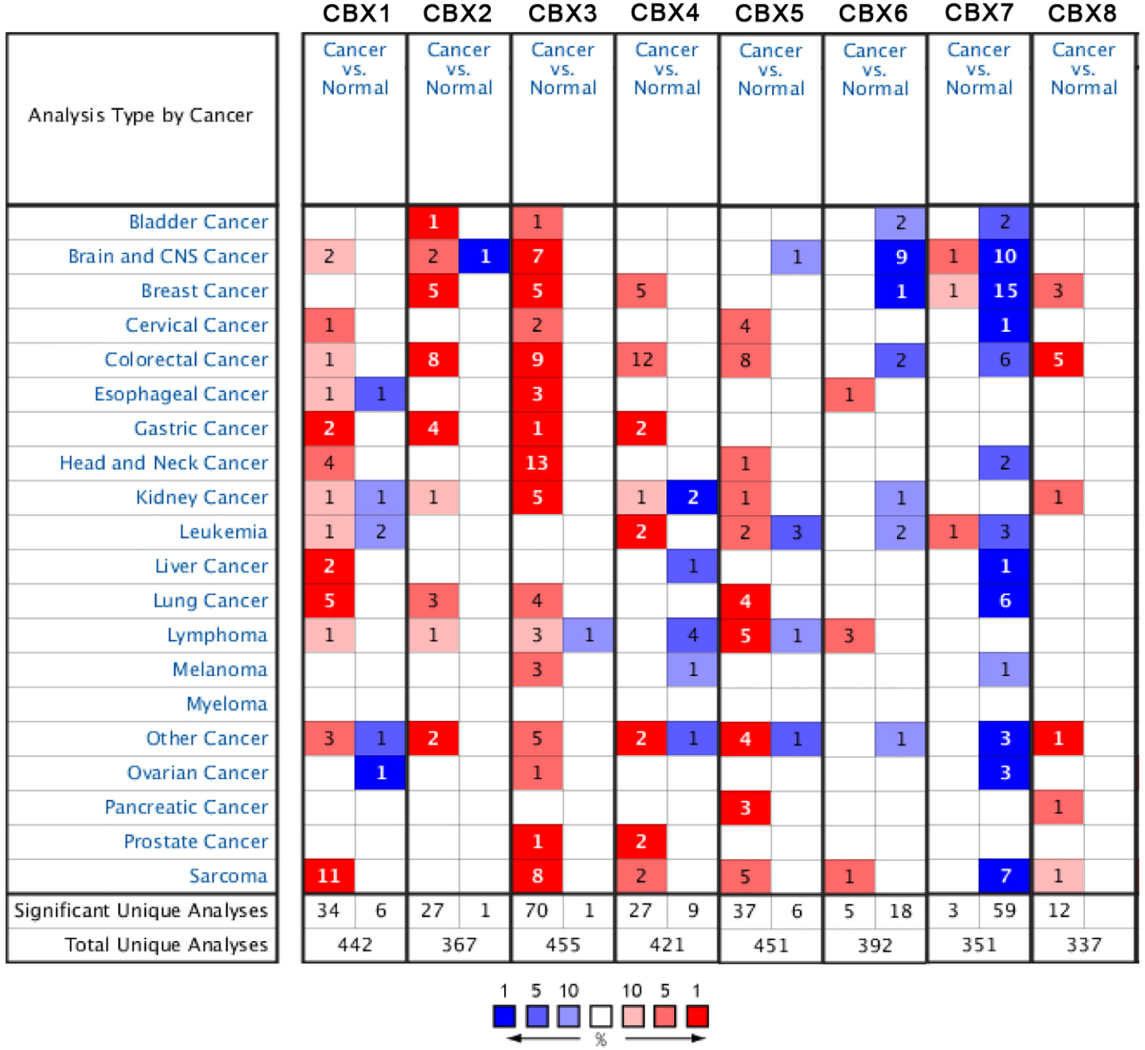
The expression levels of CBX genes in different types of human cancers and normal samples. The red cells represent evidence of gene overexpression. The blue cells represent evidence of reduced gene expression. The numbers in each cell represent the evidential frequencies. The deeper the color, the higher the significance.

**Figure 2:**
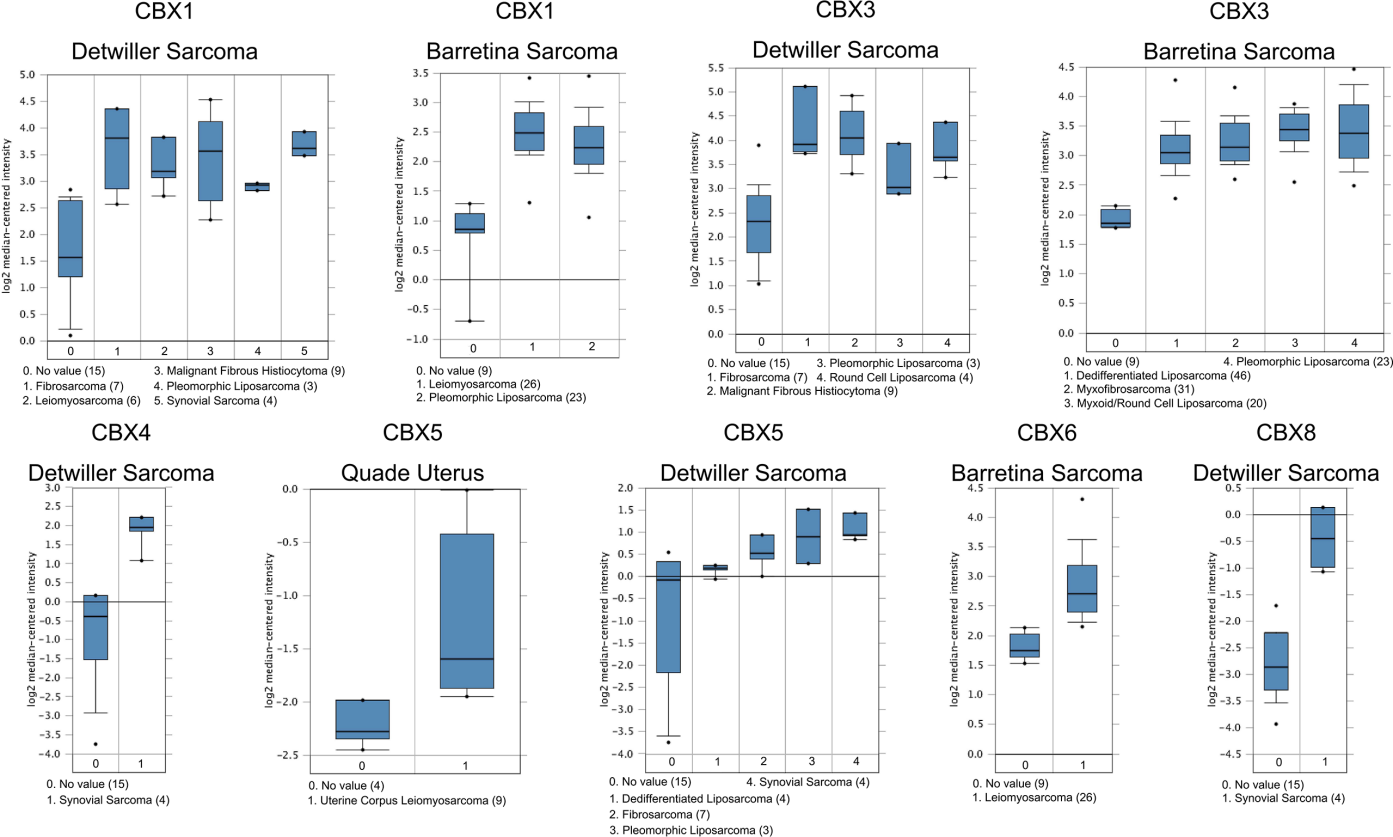
Significant changes of CBX gene expression at the transcription level between different types of sarcoma and normal tissues. The horizontal axis represents the type of tissue, and the vertical axis represents the expression level of CBX genes. The number next to the name of the sarcoma subtype represents the specific subtype *vs* ‘normal’ cases.

According to data arising from the Oncomine database, we found that HP1-*α/β/γ* (CBX1/3/5) and CBX4/6/8 were all highly expressed in sarcoma (**Figure 1**). Additionally, three databases including Detwiller sarcoma database, Barretina sarcoma database, and Quade uterus database were used to analyze the differential transcriptional expression of CBXs in subtypes of sarcoma and corresponding normal tissues. The Detwiller sarcoma database, reported by Detwiller et al. (18) identified distinctly different patterns of expression between sarcomas and normal tissues as assessed by hierarchical clustering analysis. The Barretina sarcoma database, established by Barretina et al. (19), described an integrative analysis of DNA sequence, copy number, and mRNA expression in 207 sarcoma samples encompassing seven major subtypes. The Quade uterus database, reported by Quade et al. (20), depicted RNAs profiled from four normal uterine myometria, seven uterine leiomyomas, and nine uterine leiomyosarcomas. As shown in **Figure 2**, the expression levels of HP1-*β* (CBX1) were upregulated in the Detwiller sarcoma database; the fold-changes for the expression of these genes in synovial sarcoma, pleomorphic liposarcoma, leiomyosarcoma, fibrosarcoma, and malignant fibrous histiocytoma were 4.180, 2.460, 3.155, 4.010, and 3.294, respectively. With regard to the Barretina sarcoma database, the expression levels of HP1-*β* (CBX1) in pleomorphic liposarcoma showed a fold-change of 2.826 when compared with normal samples and a fold-change of 3.315 when compared with normal samples in leiomyosarcoma.

With regard to the Detwiller sarcoma database, the fold-changes of HP1-*γ* (CBX3) expression in fibrosarcoma, malignant fibrous histiocytoma, pleomorphic liposarcoma, and round cell liposarcoma were 3.515, 3.249, 2.242, and 2.645, respectively. The expression levels of HP1-*γ* (CBX3) in dedifferentiated liposarcoma, myxoid/round cell liposarcoma, myxofibrosarcoma, and pleomorphic liposarcoma were upregulated by 2.270, 2.803, 2.439, and 2.785, fold for the Barretina sarcoma database. The fold-change for CBX4 expression was 6.516 in synovial sarcoma when compared with normal tissues for the Detwiller sarcoma database. High expression levels of HP1-*α* (CBX5) were found in uterine corpus leiomyosarcoma, synovial sarcoma, pleomorphic liposarcoma, dedifferentiated liposarcoma, and fibrosarcoma, with fold-changes of 2.005, 3.713, 3.401, 2.018, and 2.673, respectively (Quade uterus database and Detwiller sarcoma database). The expression levels of CBX6/8 in leiomyosarcoma (Barretina sarcoma database) and synovial sarcoma (Detwiller sarcoma database) were raised by 2.068- and 5.159-fold, respectively.

### The mRNA Levels of CBX Genes in Sarcoma

**Figure 3:**
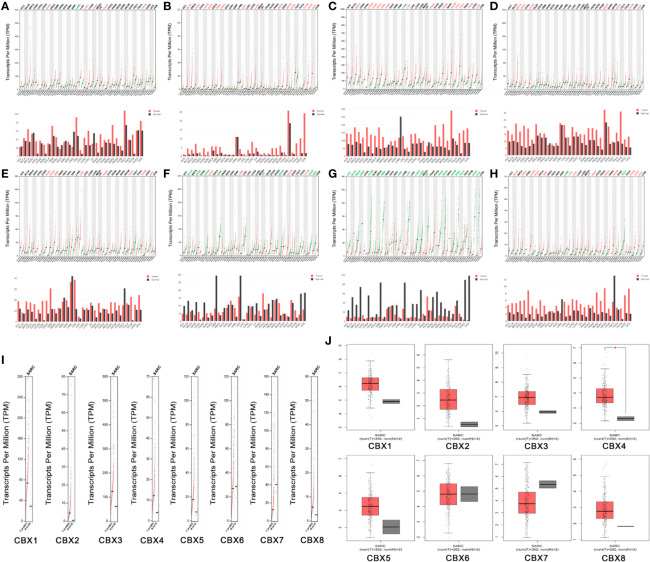
The expression levels of CBX genes in sarcoma. **(A–H)** The expression levels of CBX1-8 in pan-cancer, **(I–J)** The expression levels of CBX genes in sarcoma. The expression levels of CBX genes in sarcomas. Each dot represents an individual sample, ^*^P < 0.05.

Next, we used the GEPIA dataset to compare the expression of CBX family mRNAs between sarcoma and normal tissues. The mRNA levels of HP1-*α/β/γ* (CBX1/3/5) and CBX2/4/8 were significantly higher than those in normal tissues. In contrast, the mRNA expression levels of CBX6/7 in sarcoma were lower than those in normal tissues (**Figure 3**).

### The Expression Levels of CBX Genes in Sarcoma Cell Lines

**Figure 4:**
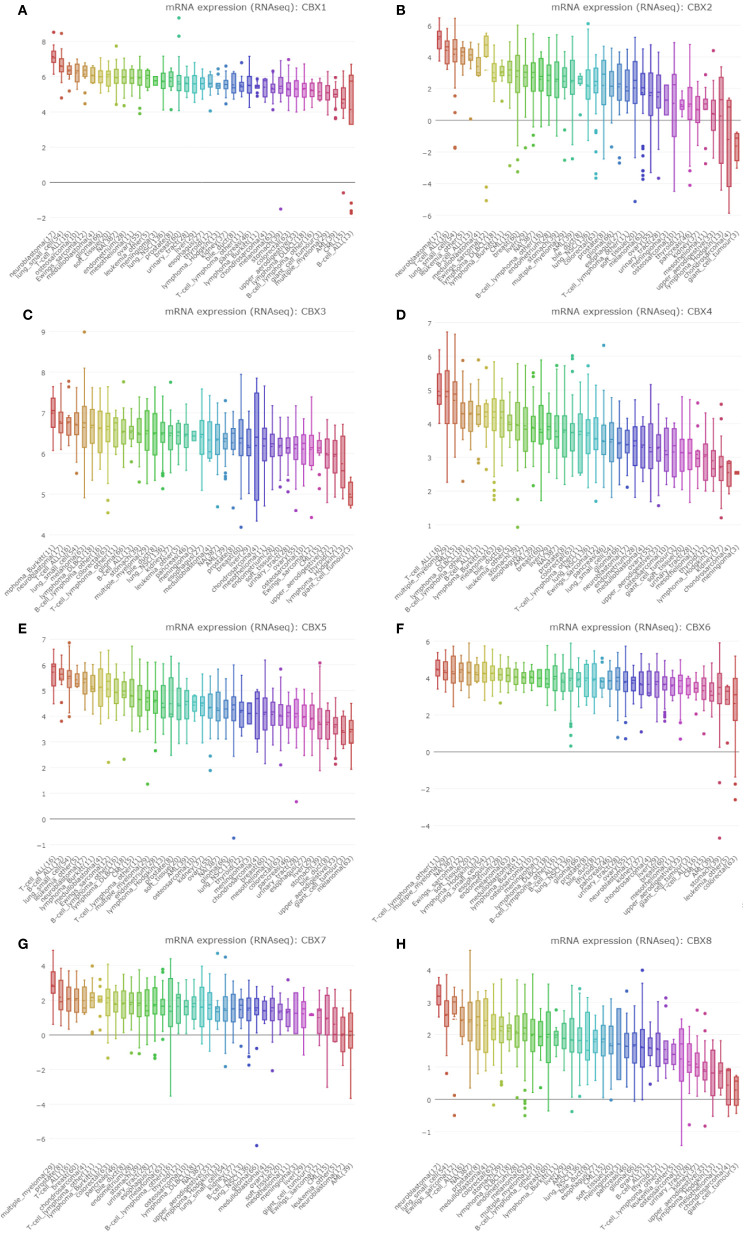
The expression of CBXs in sarcoma cell lines. **(A–H)** The expression of CBX1-8 in sarcoma cell lines. The number next to the lineage name represents number of cell lines in the lineage. The dashed line within a box is the mean.

We used the CCLE dataset to investigate the expression levels of CBXs in human cancer. Data showed that all eight members of the CBX family were expressed at high levels in sarcoma cell lines (**Figure 4**).

### The Prognostic Value of CBXs in Sarcoma

**Figure 5:**
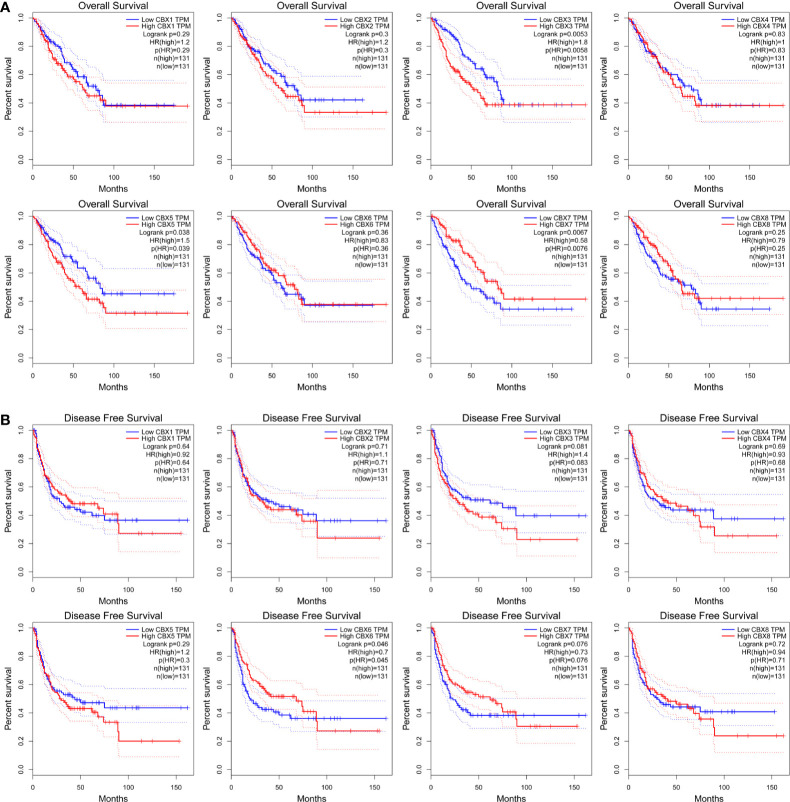
The prognostic significance of CBXs in sarcoma patients. **(A)** The association between the elevated expression of CBX genes and overall survival in patients with sarcoma. **(B)** The association between high expression levels of CBX genes and disease-free survival in patients with sarcoma. HR, hazard ratio; TPM, Transaction per million.

**Figure 6:**
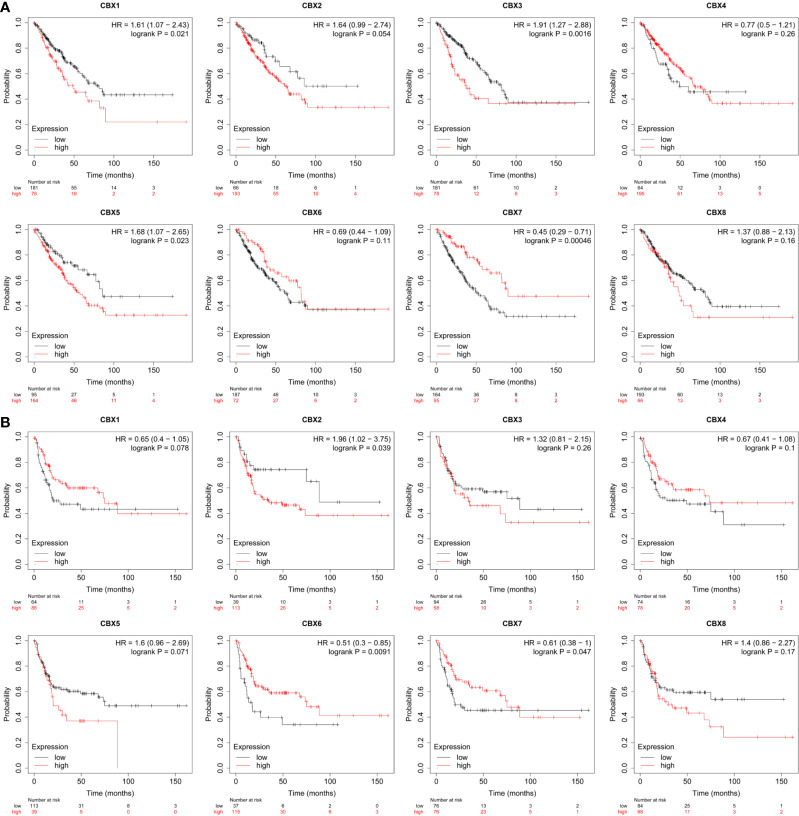
The prognostic value of CBX genes in sarcoma patients. **(A)** The association between high expression levels of CBX genes and the overall survival of patients with sarcoma. **(B)** The association between elevated expression levels of CBX genes and the recurrence-free survival of patients with sarcoma. HR, hazard ratio.

The prognostic value of CBX gene expression in sarcoma was evaluated by GEPIA and the Kaplan**–**Meier Plotter Database. As shown in **Figure 5** (GEPIA), increased expression levels of HP1-*α/γ* (CBX3/5) were associated with a poorer OS in patients with sarcoma, while elevated expression levels of CBX7 were associated with a better OS. HP1-*β* (CBX1) and CBX2/4/6/8 also tended to exert impact on the OS, but without statistical significance. High expression levels of CBX6 appear to be associated with a better DFS. Similar findings were evident in our Kaplan**–**Meier analysis (**Figure 6**). The expression levels of HP1-*α/β/γ* (CBX1/3/5) and CBX2 were associated with a poorer OS, while the levels of CBX7 were associated with a better OS. Furthermore, high expression levels of CBX6/7 were associated with a better RFS, while high expression levels of CBX2 were associated with a worse RFS.

### The Identification of Genes That Were Co-Expressed With CBX Genes in Sarcoma

**Figure 7:**
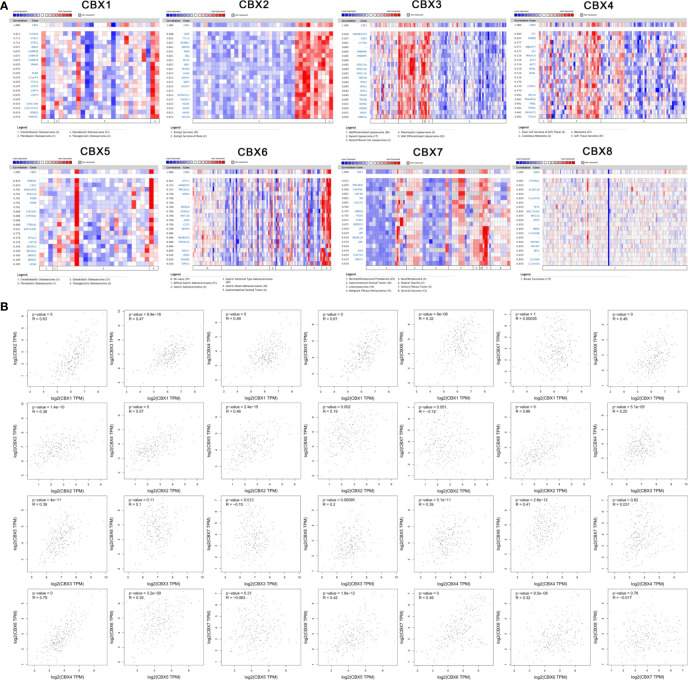
Co-expression analysis. **(A)** Genes that were co-expressed with CBX genes in sarcoma. **(B)** Correlation between CBX genes in sarcoma.

We used the Oncomine database to identify a series of genes that were co-expressed with CBX genes in sarcoma. HP1-*β* (CBX1) was co-expressed with S100A6, STAG1, MBD4, CHMP2B, PMM1, PCNP, C5orf54, TTC33, CEP70, and KIAA1586. CBX2 was co-expressed with USPS, TTLL4, ASRBK1, SMYDS, MDFI, BSG, MTA1, YBX1, MCM4, and GGA2. HP1-*γ* (CBX3) was co-expressed with HNRNPAZ2B1, CCT6A, HNRNPR, SFRS13A, DDX46, SFRS2, SFRS3, TRA2B, NONO, MSH2, and HP1-*γ* (CBX3). CBX4 was co-expressed with SF1, RABSB, MBOAT7, PRKACA, AKT2, WASL, CCNG1, UBL4A, PRKAR2A, and TRIB1. HP1-*α* (CBX5) was co-expressed with WDHD1, CDK2, ARHGAP28, PCDH19, RORB, CYP39A1, ST85IA1, PAR1GD52, SPTLC2, and ZDHHHC2. CBX6 was co-expressed with SYP11, ANKRD35, PRICKLE1, MXD4, ZBTB20, CD99L2, RNF150, JAM3, CLIP3, and MFAP4. CBX7 was co-expressed with PDCD6IP, CNOT6L, UNC80, SRI, KGFLP2, MRPS6, PCM1, S1PR1, SEPSECS, and LPP. CBX8 was co-expressed with CYP46A1, ALOX12B, C1orf109, TET2, MGC16384, MYO1A, HTR7, PNKD, C7orf28B, and ASPHD2 (**Figure 7A**).

Similarly, we analyzed the co-expression relationships between different CBX genes. HP1-*β* (CBX1) was positively correlated with CBX2 (R = 0.63, P < 0.05), HP1-*γ* (CBX3) (R = 0.47, P < 0.05), CBX4 (R = 0.49, P < 0.05), HP1-*α* (CBX5) (R = 0.61, P < 0.05), CBX6 (R = 0.32, P < 0.05), and CBX8 (R = 0.49, P < 0.05), but there was no significant correlation between HP1-*β* (CBX1), and CBX7. CBX2 was significantly correlated with HP1-*γ* (CBX3) (R = 0.38, P < 0.05), CBX4 (R = 0.57, P < 0.05), HP1-*α* (CBX5) (R = 0.46, P < 0.05), CBX6 (R = 0.19, P < 0.05), and CBX8 (R = 0.66, P < 0.05). HP1-*γ* (CBX3) was significantly correlated with CBX4 (R = 0.25, P < 0.05), HP1-*α* (CBX5) (R = 0.39, P < 0.05), CBX7 (R = −0.15, P < 0.05) and CBX8 (R = 0.2, P < 0.05). CBX4 significantly correlated with HP1-*α* (CBX5) (R = 0.35, P < 0.05), CBX6 (R = 0.41, P < 0.05), and CBX8 (R = 0.79, P < 0.05). HP1-*α* (CBX5) was significantly correlated with CBX6 (R = 0.35, P < 0.05) and CBX8 (R = 0.42, P < 0.05). In addition, CBX6 was significantly correlated with CBX7 (R = 0.49, P < 0.05) and CBX8 (R = 0.32, P <0.05). However, there was no significant correlation between CBX7 and CBX8 (**Figure 7B**).

**Figure 8:**
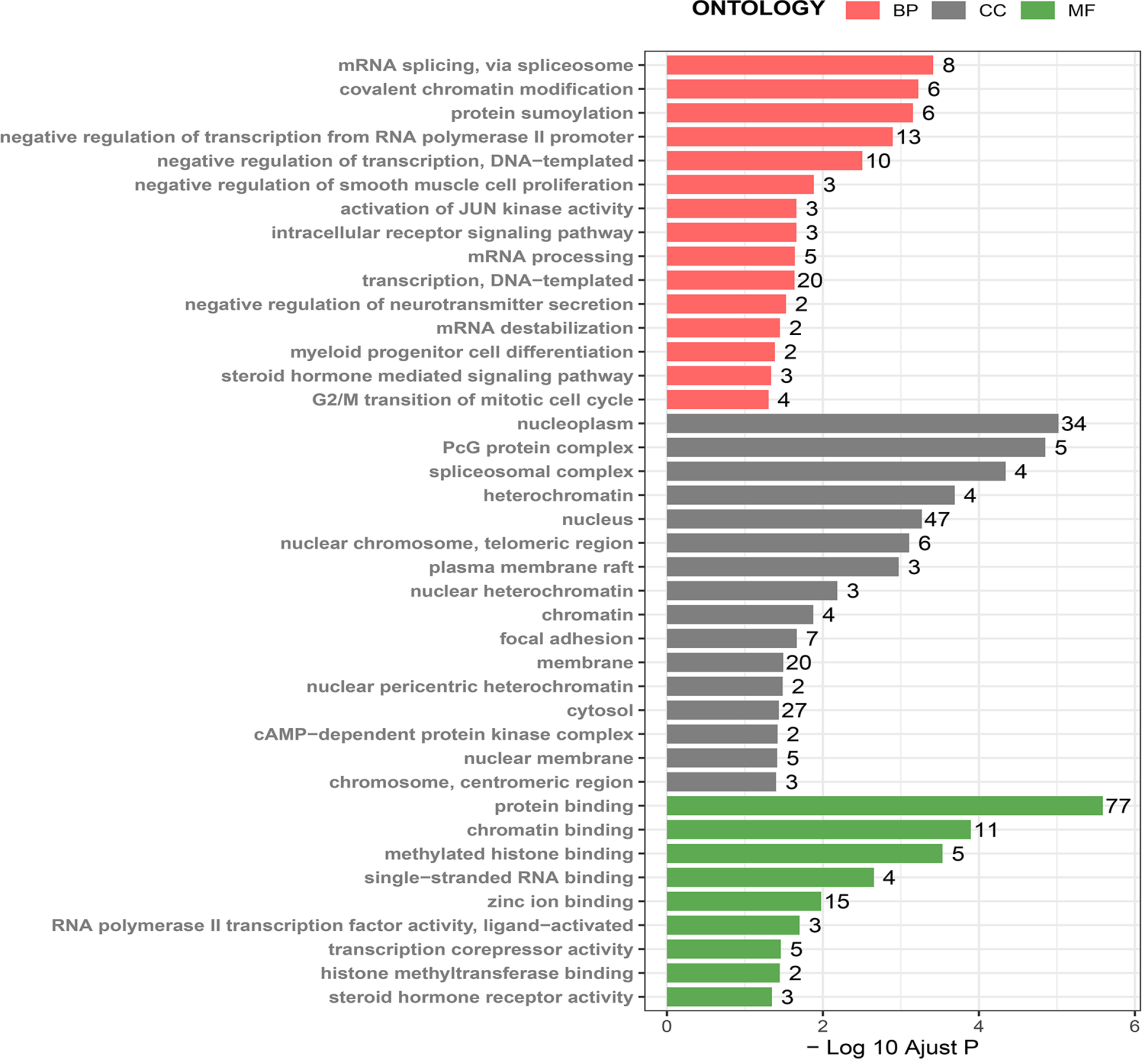
GO analysis of CBX genes and co-expressed genes. GO analysis was based on three aspects: (BP) biological processes, (CC) cellular components, and (MF) molecular function.

Next, we used the DAVID online tool to perform GO function enrichment analysis from three aspects: biological processes (BPs), cellular components (CCs), and molecular functions (MFs). We found that CBXs and their co-expressed genes were mainly involved in GO:0000398 (mRNA splicing, *via* spliceosome) for BP, GO:0005634 (nucleus) for CC, and GO:0003682 (chromatin binding) for MF. More detailed information is given in **Figure 8** and **Table 1**.

**Table 1:** GO analysis of CBXs and the co-expression genes.

The enriched	GO-ID	Description	Count	P-Value	Genes
BP	0000398	mRNA splicing, *via* spliceosome	8	3.86E-04	DDX46, TRA2B, NONO, SRRT, HNRNPA2B1, HNRNPR, YBX1, SF1
BP	0016569	covalent chromatin modification	6	5.98E-04	CBX7, CBX6, SMARCD3, CBX4, CBX3, CBX2
BP	0016925	protein sumoylation	6	7.01E-04	CBX8, STAG1, MTA1, CBX4, KIAA1586, CBX2
BP	0000122	negative regulation of transcription from RNA polymerase II promoter	13	0.001273	CBX8, CBX7, CBX6, KDM1A, CBX4, CBX2, NR2F1, ZBTB20, YBX1, NR2F2, HNRNPA2B1, MDFI, MXD4
BP	0045892	negative regulation of transcription, DNA-templated	10	0.003125	CBX5, KDM1A, CBX4, CBX3, NONO, CBX1, RORB, NR2F2, PRICKLE1, MDFI
BP	0048662	negative regulation of smooth muscle cell proliferation	3	0.013084	VIPR2, TRIB1, SF1
BP	0030522	intracellular receptor signaling pathway	3	0.021878	NR2F1, RORB, NR2F2
BP	0007257	activation of JUN kinase activity	3	0.021878	PTPN1, ZAK, MDF
BP	0006397	mRNA processing	5	0.022920	CNOT6L, NONO, HNRNPA2B1, HNRNPR, PRKACA
BP	0006351	transcription, DNA-templated	20	0.023107	CBX8, CBX7, CBX6, SMARCD3, CNOT6L, KDM1A, CBX4, CBX3, NONO, CBX2, RFX2, NR2F1, ZBTB20, WASL, RORB, NR2F2, MTA1, BRWD1, MXD4, SF1
BP	0046929	negative regulation of neurotransmitter secretion	2	0.029718	PNKD, SYT11
BP	0061157	mRNA destabilization	2	0.035555	CNOT6L, HNRNPR
BP	0002318	myeloid progenitor cell differentiation	2	0.041358	TET2, JAM3
BP	0043401	steroid hormone mediated signaling pathway	3	0.046159	NR2F1, RORB, NR2F2
BP	0000086	G2/M transition of mitotic cell cycle	4	0.049691	PCM1, CEP70, CDK2, PRKACA
CC	0005654	nucleoplasm	34	9.53E-06	SMARCD3, KDM1A, SRRT, ZBTB20, HNRNPR, RORB, YBX1, SRI, DCAF7, MTA1, EXOSC4, AKT2, TRA2B, CEP70, PRKACA, CBX8, CBX7, WDHD1, MBD4, CBX6, CBX5, CBX4, NONO, CBX2, CBX1, NR2F1, ARHGAP28, STAG1, MSH2, CCNG1, HNRNPA2B1, CDK2, MCM4, SF1
CC	0031519	PcG protein complex	5	1.41E-05	CBX8, CBX7, CBX6, CBX4, CBX2
CC	0035102	spliceosomal complex	4	4.54E-05	CBX8, CBX7, CBX4, CBX2
CC	0000792	heterochromatin	4	2.04E-04	CBX8, CBX7, CBX6, CBX2
CC	:0005634	nucleus	47	5.39E-04	SMARCD3, DDX46, KDM1A, ZAK, ZBTB20, WASL, RORB, PRICKLE1, YBX1, LPP, PCNP, MTA1, EXOSC4, AKT2, TRA2B, PRKACA, UBL4A, CBX8, CBX7, MBD4, CBX6, CNOT6L, CBX5, CBX4, CBX3, NONO, CBX2, CBX1, TET2, RFX2, NR2F1, NR2F2, GDAP1, SEPSECS, PNKD, STAG1, HNRNPA2B1, CDK2, CHMP2B, S100A6, MCM4, TRIB1, BRWD1, MDFI, TSPYL5, MXD4, SF1
CC	0000784	nuclear chromosome, telomeric region	6	7.84E-04	CBX5, MSH2, KDM1A, CBX3, CBX1, MCM4
CC	0044853	plasma membrane raft	3	0.001067	MYO1A, PRKAR2A, PRKACA
CC	0005720	nuclear heterochromatin	3	0.006535	CBX5, CBX3, CBX1
CC	0000785	chromatin	4	0.013301	MBD4, STAG1, CBX3, CBX1
CC	0005925	focal adhesion	7	0.021639	PDCD6IP, STX16, PRKAR2A, BSG, NHS, CD99L2, LPP
CC	0016020	membrane	20	0.032225	UBL4A, RAB5B, VIPR2, PDCD6IP, DDX46, STX16, MBOAT7, NONO, SRI, GDAP1, PNKD, PCM1, MSH2, PRKAR2A, BSG, HNRNPA2B1, MCM4, ASPHD2, PRKACA, CD320
CC	0031618	nuclear pericentric heterochromatin	2	0.032800	CBX5, CBX3
CC	0005829	cytosol	27	0.036723	STX16, RAP1GDS1, ALOX12B, WASL, PRICKLE1, SRI, PCM1, EXOSC4, PRKAR2A, AKT2, CEP70, PRKACA, NADK, ATP6V1C1, UBL4A, PTPN1, CNOT6L, PDCD6IP, PMM1, ARHGAP28, CCT6A, CLIP3, SEPSECS, STAG1, CDK2, CHMP2B, S100A6
CC	0005952	cAMP-dependent protein kinase complex	2	0.038162	PRKAR2A, PRKACA
CC	0031965	nuclear membrane	5	0.038575	PCM1, CEP70, PRICKLE2, PRICKLE1, YBX1
CC	0000775	chromosome, centromeric region	3	0.039848	STAG1, CBX3, CBX1
MF	0005515	protein binding	77	2.58E-06	KDM1A, ZAK, HNRNPR, WASL, YBX1, RORB, DCAF7, ROBO1, PCM1, AKT2, BSG, PRKACA, WDHD1, CNOT6L, PDCD6IP, USP5, TTC33, TET2, RFX2, MRPS6, CPT1B, CLIP3, MSH2, S100A6, MCM4, TRIB1, MDFI, TSPYL5, SF1, RAB5B, STX16, SRRT, RAP1GDS1, CD99L2, ALOX12B, PRICKLE1, SRI, LPP, PCNP, GGA2, NEURL1B, MTA1, EXOSC4, TRA2B, C1ORF109, PRKAR2A, CEP70, ATP6V1C1, NADK, JAM3, UBL4A, CBX8, PTPN1, CBX7, MBD4, CBX6, CBX5, CBX4, PMM1, CBX3, MBOAT7, NONO, CBX2, CBX1, NR2F1, NR2F2, CCT6A, MFAP4, SEPSECS, STAG1, PNKD, SYT11, CCNG1, HNRNPA2B1, CDK2, CHMP2B, MXD4
MF	0003682	chromatin binding	11	1.27E-04	CBX7, STAG1, MTA1, CBX5, SMARCD3, KDM1A, CBX4, NONO, CBX2, CBX1, YBX1
MF	0035064	methylated histone binding	5	2.93E-04	CBX8, CBX7, CBX5, CBX4, CBX2
MF	0003727	single-stranded RNA binding	4	0.002218	CBX8, CBX7, CBX6, CBX4
MF	0008270	zinc ion binding	15	0.010524	PTPN1, USP5, PRICKLE2, TET2, NR2F1, PRICKLE1, RORB, NR2F2, ZDHHC2, LPP, NEURL1B, MTA1, S100A6, RNF150, SF1
MF	0004879	RNA polymerase II transcription factor activity, ligand-activated	3	0.019929	NR2F1, RORB, NR2F2
MF	0003714	transcription corepressor activity	5	0.034675	MTA1, CBX4, NR2F2, MXD4, SF1
MF	1990226	histone methyltransferase binding	2	0.035716	CBX3, CBX1
MF	0003707	steroid hormone receptor activity	3	0.045081	NR2F1, RORB, NR2F2

### Immune Infiltration Levels in Sarcoma

**Figure 9:**
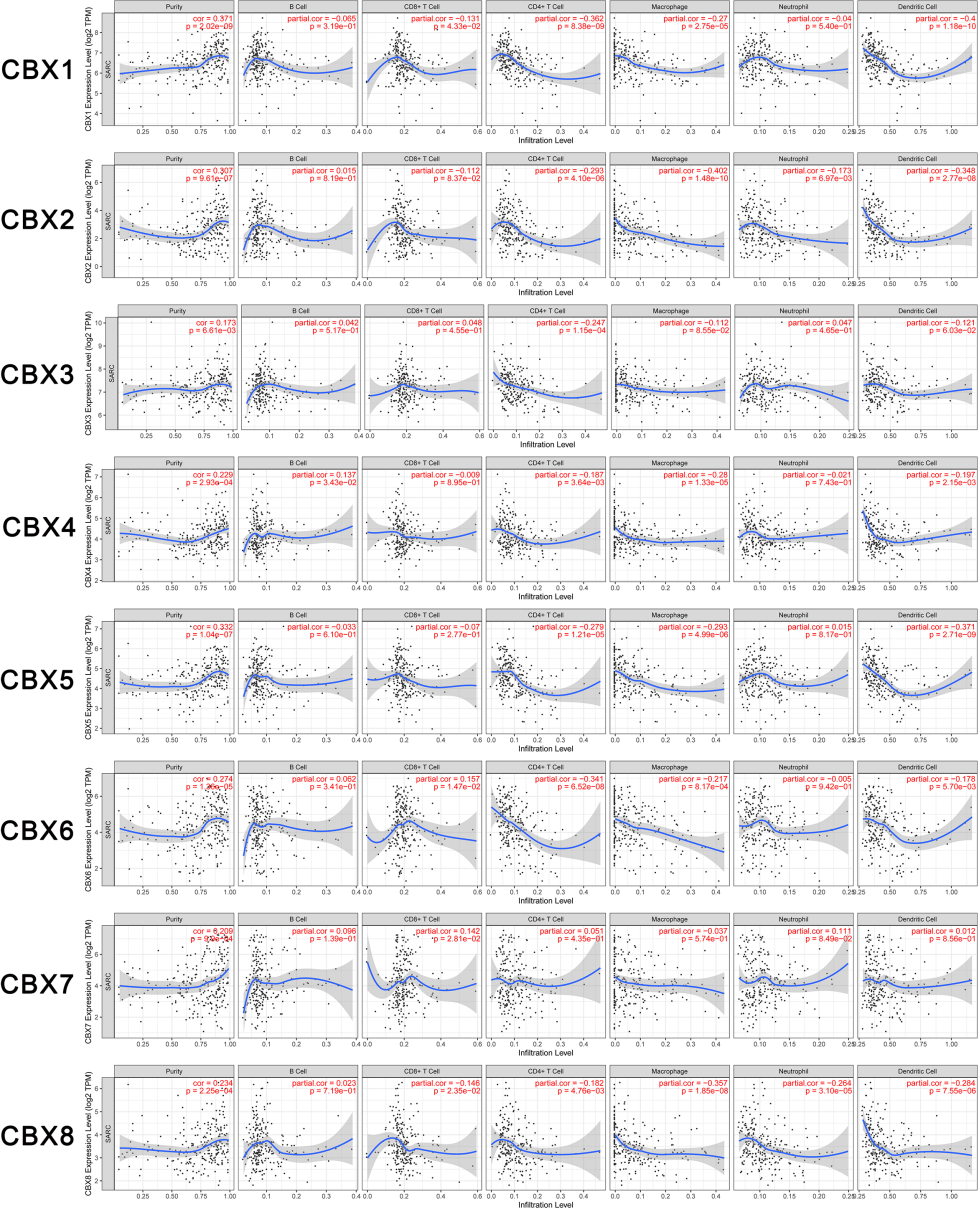
Relationship between differentially expressed CBX genes and immune cell infiltration. The immune cells we analyzed included B cells, CD8^+^ T cells, CD4^+^ T cells, macrophages, neutrophils, and dendritic cells.

In the present study, the TIMER dataset was used to analyze whether the expression of CBX genes were correlated with the immune infiltration of sarcoma. We found that HP1-*β* (CBX1) expression was significantly associated with the infiltration of CD8^+^ T cells (Correlation coefficient (cor) = −0.131, p < 0.05), CD4^+^ T cells (cor = −0.362, p < 0.05), macrophages (cor = −0.27, p < 0.05), and dendritic cells (cor = −0.4, p < 0.05). CBX2 was significantly associated with the infiltration of CD4^+^ T cells (cor = −0.293, p < 0.05), macrophages (cor = −0.402, p < 0.05), neutrophils (cor = −0.173, p < 0.05), and dendritic cells (cor = −0.384, p < 0.05). HP1-*γ* (CBX3) was significantly associated with infiltration by CD4^+^ T cells (cor = −0.247, p < 0.05). CBX4 was significantly associated with infiltration by B cells (cor = 0.137, p < 0.05), CD4^+^ T cells (cor = −0.187, p < 0.05), macrophages (cor = −0.28, p < 0.05), and dendritic cells (cor = −0.197, p < 0.05). HP1-*α* (CBX5) was significantly associated with infiltration by CD4^+^ T cells (cor = −0.279, p < 0.05), macrophages (cor = −0.293, p < 0.05) and dendritic cells (cor = −0.371, p < 0.05). CBX6 was significantly associated with infiltration by CD8^+^ T cells (cor = 0.157 p < 0.05), CD4^+^ T cells (cor = −0.341 p < 0.05), macrophages (cor = −0.217 p < 0.05), and dendritic cells (cor = −0.178 p < 0.05). CBX7 was significantly associated with infiltration by CD8^+^ T cells (cor = 0.142 p < 0.05). Finally, CBX8 was significantly associated with infiltration by CD8^+^ T cells (cor = −0.146 p < 0.05), CD4^+^ T cells (cor = −0.182 p < 0.05), macrophages (cor = −0.357 p < 0.05), neutrophils (cor = −0.264 p < 0.05), and dendritic cells (cor = −0.284 p < 0.05) (**Figure 9**).

## Discussion

The CBX family of genes is thought to represent vital elements in a variety of tumors (14–16). However, the association between CBXs and sarcoma has not been extensively reported. Currently, there is a renewed interest in immunotherapy for cancer. Most immunotherapy studies have focused on melanoma. These have revealed that the overexpression of GM2, GD2, and GD3 immunogenicity gangliosides can trigger an antibody response and improve the survival rate of patients with melanoma. Other studies have investigated MHC-restricted cancer-testicular antigens; these have also raised the prospect of immunotherapy (22). Subsequently, the concept of tumor vaccines was proposed for specific tumor epitopes by stimulating an immune response. Therefore, immune-related studies on sarcomas, combined with radiotherapy, chemotherapy, and several targeted therapies, have become increasingly popular (23). Therefore, we conducted this study to reveal the relationships between CBX genes and human sarcoma, particularly with respect to their prognostic value and the immune infiltration of CBXs.

The up-regulated expression of HP1-*β* (CBX1) has been reported for many forms of cancer. For example, Liang et al. reported high expression levels of HP1-*β* (CBX1) mRNA in breast cancer and found that this was associated with a poorer RFS. HP1-*β* (CBX1) has also been associated with chemoresistance in patients with breast cancer. Therefore, these authors defined HP1-*β* (CBX1) as a potential target for the treatment of breast cancer (14). In addition, Yang et al. found that the overexpression of HP1-*β* (CBX1) in hepatocellular carcinoma could activate the Wnt/*β*-Catenin signaling pathway by interacting with the transcription factor HMGA2, thus suggesting that HP1-*β* (CBX1) was an independent factor for hepatocellular carcinoma (24). Similar results were reported by Gang et al. (17). Higher expression levels of HP1-*β/γ* (CBX1/3) and CBX2/6/8 were reported to be related to a worse OS while higher expression levels of CBX7 were associated with a better OS. These results are similar to our present results which indicated that the up-regulated expression of HP1-*α/γ* (CBX3/5) is related to a poorer OS, while the up-regulated expression of CBX7 was associated with a better OS. Similar results were obtained from our Kaplan–Meier Plotter analyses.

According to a previous study, CBX2 is a key member of the polycomb group (PcG) family and CBX2 mRNA was predominately localized in spermatogonia and spermatocytes, as demonstrated by *in situ* hybridization (ISH) (25). Tatavosian et al. found that CBX2 undergoes phase separation to form condensates and that these CBX2 condensates concentrate DNA and nucleosomes (26). Plys et al. further reported that the domain of CBX2 was the same as the domain related to chromatin compaction and development, thus improving the possibility of an evolutionary or mechanistic link between these activities (27). In another study, Alexandra et al. reported that the stabilization of the testis required CBX2-mediated repression of bivalent ovary-determining genes (28). In hepatocellular carcinoma, the knockdown of CBX2 restrained the proliferation of HCC cells and increased the phosphorylation of YAP. These data suggest that CBX2 could be a potential target for hepatocellular carcinoma (29). In addition, Zheng et al. showed that higher expression levels of CBX2 were significantly and independently associated with a worse OS by affecting the PI3K/AKT signaling pathway in breast cancer (30). In our present study, we observed elevated expression levels of CBX2 and found that CBX2 was associated with a poorer OS, thus indicating that this gene might be an independent prognostic factor for human sarcoma.

High expression levels of HP1-*γ* (CBX3) [encoded by HP1-*γ* (CBX3)] are known to accelerate HCC cell proliferation, thus suggesting that HP1-*γ* (CBX3) is a crucial oncogene in hepatocellular carcinoma (31). Similar findings have been reported for glioma; high expression levels of HP1-*γ* (CBX3) are known to predict a worse prognosis (32). In gastric cancer, Lin et al. found that HP1-γ (CBX3) was overexpressed and could regulate genes associated with the cell cycle, mismatch repair, and immune-related pathways. The expression level of HP1-*γ* (CBX3) was also significantly and inversely related to the expression levels of TILs, PDCD1, and PDCD2, and immunotherapy responses, thus implying that HP1-*γ* (CBX3) could influence the efficacy of immunotherapy and chemotherapy (33). Ma et al. further confirmed the function and role of HP1-*γ* (CBX3) in osteosarcoma, by showing that the expression of HP1-*γ* (CBX3) was associated with a poorer DFS and OS, as well as a larger tumor size, a higher distant metastasis rate, and a higher clinical stage. These authors used HP1-*γ* (CBX3) siRNA to knockdown HP1-*γ* (CBX3) and thus block proliferation ability, thus resulting in increased levels of apoptosis and cell cycle arrest at the G0 and G1 phase (12); these findings were consistent with those reported in the present study. Another study reported that the elevated expression of HP1-*γ* (CBX3) was related to unfavorable OS in patients with human sarcoma. CBX4 is known to recruit GCN5 to the Runx2 promoter to transcriptionally upregulate Runx2; in this manner, CBX4 can promote the metastasis of osteosarcoma (13). In another paper, Hu et al. reported that the cell growth and migration of human lung cancer cell was suppressed by the knockdown of CBX4, both *in vitro* and *in vivo*. Furthermore, CBX4 has been shown to promote proliferation and metastasis by regulating the BMI-1 pathway, thus suggesting that CBX4 might be a potential therapeutic target in lung cancer (34). In the digestive system, the suppression of Runx2 by CBX4 resulted in the inhibition of cell migration, invasion, and metastasis (35). Interestingly, Ren et al. reported that CBX4 counteracts senescence in human mesenchymal stem cells (HMSC) by maintaining nucleolar homeostasis, and that CBX4 maintained nucleolar homeostasis by recruiting nucleolar protein-fibrillin and heterochromatin KRAB associated protein 1 (KAP1) within nucleolar rDNA, thereby limiting rRNA overexpression and attenuating the development of osteoarthritis in mice (36).

In the present study, we found that CBX4 was highly expressed in human sarcoma tissues. Guo et al. were the first to confirm the high expression levels of HP1-α (CBX5) in gastric cancer tissues, and then revealed that HP1-*α* (CBX5) could promote the proliferation, migration, and invasion, of gastric cancer cells *in vitro* (37). In lung cancer, Yu et al. used a panel of tumor stem-like cells (hESCs) to verify the materiality of HP1-*α* (CBX5) (38). In our study, increased levels of HP1-*α* (CBX5) expression were related to a poorer OS, both in the GEPIA dataset and the Kaplan–Meier Plotter dataset. Wang et al. reported that the level of anti-HP1-*α* (CBX5) antibody was associated with age, cigarette-smoking habits, and blood pressure, in patients who had suffered from transient ischemic attack, thus indicating that serum levels of antibodies against HP1-*α* (CBX5) could potentially serve as tools for diagnosing transient ischemic attack (39). The elevated expression of CBX6 has been reported in HCC tissues and cell lines; CBX6 was also correlated with a larger tumor size (≥5 cm; p = 0.011). Moreover, HCC patients with higher expression levels of CBX6 showed a worse OS and RFS than patients with lower expression levels of CBX6 (40). Deng et al. identified CBX6 as a downregulated gene in breast cancer *via* a comprehensive analysis of The Cancer Genome Atlas (TCGA); their final findings supported CBX6 as a cancer suppressor in breast cancer (41). Similarly, our present study suggested that higher expression levels of CBX6 were related to DFS in patients with sarcoma.

Reduced expression levels of CBX7 have been found to be associated with a poorer OS and the aggressiveness of thyroid cancers, colorectal cancers and breast cancers (42–44), In HCC, the downregulation of CBX7 may be related to a short OS (17). However, high expression levels of CBX7 were associated with a reduced OS and DFS in patient with prostate cancers and ovarian cancers (45, 46). In sarcoma, we found that the expression level of CBX7 was downregulated in sarcoma, and that the elevated expression of CBX7 was associated with a better OS and DFS. Therefore, further studies are still needed to fully evaluate the role of CBX7 in cancers. CBX8 was found to have the ability to promote invasion and migration in breast cancer, lung cancer, and glioblastoma (47), and in HCC. A previous study showed that CBX8 could act as an oncogene and play an important role in upregulating the Akt/*β*-catenin pathway stimulated by EGR1 and miR-365-3p. The upregulated expression of CBX8 was also correlated with a poorer OS (48). In our study, high expression levels of CBX8 tended to exert impact on the survival rate but without statistical significance.

The tumor microenvironment can affect the progression and recurrence of multiple cancers. Immune cells within the tumor microenvironment have been shown to promote or suppress cancer activities and are considered as an important determining factor in clinical outcome and immune therapy. In the present study, we found that the expression levels of CBX genes were significantly related to immune cell infiltration, thus indicating that CBXs could reflect the immune status of sarcoma. Our study can provide more detailed immune information to sustain immune therapy for patients with sarcoma. Our study has several limitations that need to be considered. First, the data used for analysis were obtained from online services. We need to carry out more cell-based studies and clinical experiments to confirm our findings and to further explore interactions between relevant molecules, the precise mechanisms involved, and the potential clinical applications of CBX genes in sarcoma. In addition, the performance of immune infiltration should be validated by co-localization with different members of the CBX family and immune infiltration markers.

## Conclusion

In conclusion, our study showed that HP1-*α/β/γ* (CBX1/3/5) and CBX4/6/8 were highly overexpressed in human sarcoma tissues. The high expression levels of HP1-*α/γ* (CBX3/5) were closely associated with a poorer OS while the high expression levels of CBX7 were associated with a greater OS. CBX genes were positively correlated with the infiltration of immune cells, including CD8^+^ T cells, CD4^+^ T cells, B cells, macrophages, neutrophils, and dendritic cells, in sarcoma. These results indicated the crucial value of CBX genes in the prognosis and immune therapy of human sarcoma. Our findings may provide new insight and comprehensive analysis to select novel prognostic and immune biomarkers for sarcoma.

## Data Availability Statement

The datasets used and/or analyzed during the current study are available from the corresponding author on reasonable request.

## Author Contributions

JZ and WW conceived, designed, and conducted the experiments. JZ and ZC wrote the paper. JZ and YL collected the data. JZ and WW edited the paper. JZ and GW provided the research guide. RW, JZ, MZ, and TW revised the manuscript. YL, WW, and TL supervised this project. All authors contributed to the article and approved the submitted version.

## Funding

This work was supported by the Fundamental Research Funds for the Central Universities of Central South University (Grant No in application system. 1053320210251) and the Scientific Research Project of Hunan Health Committee (Grant No. 20200357).

## Conflict of Interest

The authors declare that the research was conducted in the absence of any commercial or financial relationships that could be construed as a potential conflict of interest.
